# Complete Genomic Characterization of Korean Porcine Epidemic Diarrhea Virus Strain KUPE21

**DOI:** 10.1128/genomeA.00105-18

**Published:** 2018-03-08

**Authors:** Jun Woo Park, Sung J. Yoo, Taeyong Kwon, Youngkwang Moon, Young S. Lyoo

**Affiliations:** a College of Veterinary Medicine, Konkuk University, Seoul, Republic of Korea

## Abstract

Nationwide porcine epidemic diarrhea virus (PEDV) outbreaks occurred in late 2013 in the Republic of Korea, resulting in an expansion of genomic data for Korean PEDVs. However, the data available for Korean PEDVs before 2013 are insufficient. Therefore, we sequenced and analyzed the complete genome of a Korean PEDV strain, KUPE21, which was isolated in the early 2000s.

## GENOME ANNOUNCEMENT

Porcine epidemic diarrhea virus (PEDV), a member of the Coronaviridae family, is a single-stranded positive-sense RNA virus that induces acute watery diarrhea with high mortality, especially in neonatal piglets. The first PEDV outbreak in the Republic of Korea was reported in 1987 (1), and several outbreaks had been reported sporadically until the early 2010s. In late 2013, nationwide PEDV outbreaks swept across the Republic of Korea (2), leading to subsequent studies and the deposit of many complete genome sequences of Korean PEDVs to public databases; however, except for some strains reported in the late 1990s, only a few complete genomes of strains isolated before 2013 were available. To supplement the genomic information for Korean PEDV strains dating to the early 2000s, we performed a retrospective study of a Korean field PEDV strain isolated in 2001. Here, we report the complete genome sequence of Korean PEDV strain KUPE21.

A fecal sample obtained in 2001 from a naturally infected piglet was processed and determined to be positive for PEDV, per the methods from a previous study (3), and the PEDV was isolated in Vero cell cultures. Total RNA was extracted from the supernatant with TRIzol reagent (Invitrogen, USA) according to the manufacturer’s protocol, and 16 overlapping cDNA fragments were amplified to encompass the complete genome, including the 5′ and 3′ ends. The purified DNA fragments were sequenced with the Sanger sequencing method (Macrogen, Inc., Republic of Korea), and the sequencing results were compared with those of complete PEDV reference genomes in GenBank.

The full-length sequence of PEDV strain KUPE21 was 28,038 nucleotides (nt) in length, excluding the 3′-end poly(A) tail. The genomic arrangement and corresponding nucleotide positions were as follows: open reading frame 1a (ORF1a), nt 293 to 12601; ORF1b, nt 12601 to 20637; spike gene, nt 20634 to 24794; ORF3, nt 24794 to 25468; envelope gene, nt 25449 to 25679; membrane gene, nt 25687 to 26367; and nucleocapsid gene nt 26379 to 27704. The complete genome of KUPE21 and those of other PEDV strains available in GenBank shared nucleotide identities that ranged from 95% to 98%, the highest of which (98%) was shared with the Chinese strain JS-HZ2012 (GenBank accession number KC210147). Comparing the complete genome of KUPE21 to that of JS-HZ2012, the nucleotide identity of each ORF was as follows: 98.7% for ORF1a, 99.2% for ORF1b, 96.1% for the spike gene, 98.6% for ORF3, 96.5% for the envelope gene, 97.6% for the membrane gene, and 96.9% for the nucleocapsid gene, respectively.

To our knowledge, this is the first complete genome of a Korean field PEDV strain from 2001 to be identified. The PEDV KUPE21 sequence data will provide useful genetic information about endemic Korean PEDVs predating the 2013 outbreaks, facilitate future investigations on the epidemiology and evolution of PEDVs in the Republic of Korea, and further illuminate our comprehension of the genetic diversity of PEDVs.

### Accession number(s).

The KUPE21 sequence data have been deposited in GenBank under the accession number MF737355.
